# Nursing Students’ Experiences of Medical Simulation: A Mixed-Method Study

**DOI:** 10.1177/23779608251318951

**Published:** 2025-02-13

**Authors:** Camilla Ingrid Eide, Ann-Sofie Magnusson, Inger Jansson

**Affiliations:** 174416 Institute of Health and Care Science, Sahlgrenska Academy, University of Gothenburg, Gothenburg, Västra Götaland County, Sweden

**Keywords:** simulation training, nursing students, acute care, crisis resource management, crew resource management, questionnaire, thematic analysis, mixed-method

## Abstract

**Introduction:**

Nursing students need to be prepared for the realities of acute situations or crises. Medical simulation training is a well-known pedagogical method for teaching acute care and teamwork. Despite that, there is a lack of knowledge about students’ experiences with the training. Such knowledge could contribute to the development of the training program.

**Objective:**

To describe nursing students’ experiences of medical simulation.

**Design:**

Mixed-method.

**Method:**

A total of 175 nursing students in semesters three and five answered a questionnaire with quantitative questions and open-ended comments. The qualitative data from the open-ended questions were analyzed inductively using thematic analysis. Quantitative data from the closed-ended questions were analyzed using descriptive statistics. Finally, a mixed-method synthesis was conducted, in which the findings from the qualitative analysis guided the synthesis, while the quantitative data supported the themes and sub-themes that emerged from the qualitative analysis.

**Result:**

An overall theme in the results emerged, which was: “Students need and want more simulation in their curriculum.” This theme was then divided into five sub-themes: “Well-structured model,” “Being prepared by practical training,” “Enhanced knowledge,” “Reflection gives self-awareness,” and “Feelings of fun and positive nervousness.”

**Conclusions:**

The result highlights medical simulation as a reliable pedagogic method because it was a well-structured model which made them prepared, gave them enhanced knowledge, and helped their self-reflection. Students recognize the need for additional medical simulation training and express a desire for both longer sessions and more opportunities. They believe that simulation training is beneficial for their future roles as nurses because it enhances their competence in acute care and improves their teamwork skills. Bloom's taxonomy is a valuable framework for designing and developing curricula, particularly when medical simulation plays a key role in achieving all levels of high cognitive skills.

## Introduction

Becoming a professional nurse and working efficiently during crises requires knowledge, experience, and practice. Transitioning from a new graduate to a working nurse is complex, and many factors are affecting new nurses, such as organizational challenges, professional challenges, and their personal lives. The transition shock experienced by new graduates needs to be minimized (Graf et al., 2020; Hawkins et al., 2019). Education can play a crucial role in alleviating professional challenges. Undergraduate nursing students must be trained to respond to various medical crises and to work effectively in teams within clinical environments (Graf et al., 2020; Willman et al., 2020). One method for nursing students to learn acute care and teamwork is medical simulation training. Simulation training allows students to reflect on their own needs for knowledge (Abelsson & Bisholt, 2017).

Medical simulation is also referred to as simulation-based training (SBT) or high-fidelity simulation (HFS). While the terminology is used inconsistently in simulation literature, these terms generally refer to training that focuses on assessment needs, formatting purposes, measurable outcomes, and uniquely realistic scenarios. As a result, the terms are often used interchangeably (Carey & Rossler, 2023).

The medical simulation uses a computer-based manikin as a patient, and teachers can program patient scenarios before their students begin the exercise. Medical simulation has been utilized for many years as a safe and experiential training method to prepare nursing students for providing acute care during crises (Hanshaw & Dickerson, 2020). Management of acute care uses the Airway, Breathing, Circulation, Disability and Exposure/Environment algorithm—also known as ABCDE. The algorithm was initially introduced as a trauma education program called Advanced Trauma Life Support (ATLS), and it has been used in medical training settings worldwide since 1988 (American College of Surgeons, 2018). This algorithm is applicable even outside trauma situations and should be utilized in any acute clinical setting (Thim et al., 2012).

There are different programs for students or professional healthcare workers to provide crisis-oriented team training. The principles of crisis/crew resource management (CRM) have originally been a training method for aviation, called crew or cockpit resource management (Walker & Youngblood, 1989). Healthcare then made an adaptation from aviation where the same principles are used. According to healthcare, CRM started with the theme of anesthesiology (Howard et al., 1992) and has been spread to all facilities of care and even into less acute settings of care for education during medical simulations. CRM is about teamwork behavior, resource management, leadership, and communication (Gaba, 2010). Lei and Palm (2023) wrote about the importance of undergoing simulation-based CRM training because the training improves cognitive behavior and interaction in the team.

Medical simulation is an expensive and time-consuming component of the curriculum, making it essential for teachers to evaluate its significance for students’ learning. Simulation can be utilized not only for learning but also for formative and summative evaluations to assess clinical competence (Felix & Simon, 2024). When designing the curriculum, teachers must plan simulations to address the specific competence domains they aim to support. According to Chiniara et al. (2012), these domains include both technical skills and nontechnical skills, such as communication, teamwork, leadership, and clinical decision-making. Teachers must also carefully consider post-scenario debriefing and the creation of supportive learning environments.

The development of the curriculum also involves teachers setting clear and measurable goals. By incorporating the six levels of Bloom's taxonomy (Adams, 2015), they can ensure a structured approach to learning, progressing from basic knowledge acquisition to deeper understanding and application. Bloom's taxonomy identifies six cognitive levels: (1) Knowledge, (2) Comprehension, (3) Application, (4) Analysis, (5) Synthesis, and (6) Evaluation (Adams, 2015). In planning medical simulation activities, teachers can leverage Bloom's taxonomy to build upon learners’ existing knowledge and skills, guiding them toward more advanced learning outcomes (Felix & Simon, 2024).

## Review of Literature

Medical simulation is recognized as a valuable and effective approach for enhancing decision-making skills and improving the ability to detect patient deterioration (Theobald et al., 2021). It has been found that medical students’ anxiety decreases after simulation training, making them feel more confident in clinical settings (Yu et al., 2021). Using a standardized scenario gives students the opportunity to train for specific crises and makes evaluation easier (Pacheco Granda & Salik, 2023). It allows students to undertake the exercise without harming a real patient, leaving them feeling confident and challenged simultaneously. After the scenario, there is an immediate feedback session for students to evaluate themselves, their team, and their choices (Ahmad et al., 2023). In this context, the feedback session, called debriefing, is led by a teacher known as a facilitator. Research on medical simulation training highlights the importance of feedback in enhancing learning. Teachers play a key role in providing feedback that allows teams to reflect on and analyze their actions during scenarios, ultimately improving their skills and preparedness for clinical practice (Ahmad et al., 2023). Debriefings typically yield more positive outcomes than criticism, as a positive perspective enables learning from more than failures (Dieckmann et al., 2017). While these positive outcomes significantly enhance learning, an overabundance of positivity can sometimes overshadow valuable criticism. Students sometimes need and even welcome constructive criticism, but negative feedback can carry the risk of offending someone (Abelsson & Bisholt, 2017). Teachers face the challenge of promoting debriefing sessions that allow students to reflect on and further analyze their experiences from the simulation (Abulebda et al., 2022). Therefore, facilitator training is recommended to optimize students’ learning outcomes and address their physiological needs within a safe learning environment (Abulebda et al., 2022; Ahmad et al., 2023).

Nursing students need to be prepared for the real acute situations or crises they will encounter as professional nurses. One way of training nursing students is through medical simulation, with crisis scenarios, working in a team, and following ABCDE and CRM principles. This specific simulation training needs evaluation of what the students’ experiences are, what they have learned, and if it makes them more prepared for an acute situation to further develop the training.

The aim of this study was to describe nursing students’ experiences of medical simulation.

## Methods

### Design

This study employed a mixed-method design, combining thematic analysis of qualitative data and descriptive statistics for quantitative data in a mixed-method synthesis.

Students from semester three and semester five in a bachelor's nursing program participated by completing a questionnaire containing both closed-ended and open-ended questions following a simulation exercise. The qualitative data from the open-ended questions were analyzed using thematic analysis (Braun & Clarke, 2021). This method was chosen because it is well suited for analyzing texts and allows for inductive exploration of experiences and perceptions, including related attitudes and how various themes are interconnected (Herzog et al., 2019).

The quantitative data from the closed-ended questions were analyzed using descriptive statistics. Finally, a mixed-method synthesis was conducted by combining and interpreting the qualitative and quantitative results (Schoonenboom & Burke Johnson, 2017). Here, the results of the qualitative analysis guided the mixed-method synthesis, and the quantitative data were used to strengthen the findings from the qualitative analysis, that is, the themes and subthemes that emerged from the qualitative analysis.

### Setting

The study was conducted on students in a three-year nursing program with a bachelor's degree at a university in western Sweden. In semesters three and five, students at the university had a course with acute care ABCDE and CRM principles as lectures, followed by a training scenario with a full-scale medical simulation.

In semester three, groups of six to eight students participated in three different scenarios where they assessed and treated a deteriorating patient (represented by a manikin). Only half of each group engaged in treating the manikin, while the other half observed. In semester five, groups of six to eight students encountered four different and more challenging scenarios, again involving patient assessment and treatment using a manikin. Overall, each student had the opportunity to actively participate in one to two scenarios and observe two scenarios.

The simulation training for both semesters was conducted over 4 hr and led by two teachers educated in acute care and teamwork. After each scenario, a debriefing session was held, including a review of the scenario, a reflection phase, and an analysis phase (Abulebda et al., 2022; Ahmad et al., 2023). During the debriefing, the students, together with the whole group of six to eight participants, analyzed what occurred with the patient, the teamwork involved, and areas for improvement. Following the debriefing session, the students were asked to complete a questionnaire.

### Sample and Data Collection

From February to May in year 2019, 72 out of 73 nursing students (99%) from semester three, who conducted the simulation, answered a questionnaire about medical simulation. From September to December in year 2019, 103 out of 105 nursing students (98%) from semester five, who conducted the simulation, completed the same questionnaire. Both semesters completed the questionnaires immediately after their group's simulation training session, which was handed out on paper by the teacher. The questionnaire took about 5 min to complete. The inclusion criterion was that students had participated in the medical simulation training class. Only one student in semester three and two students in semester five did not complete the questionnaire. There were no exclusion criteria.

Only one student (of 72) in semester three had previously undergone full-scale simulation training and only one student (of 103) in semester five had not previously undergone full-scale simulation training.

The questionnaire was developed in 2005 by the Swedish Armed Forces Centre for Defence Medicine, Gothenburg, and was used by the center after medical simulation training for at least 10 years. The questionnaire consisted of 10 questions. Questions one and two included questions about their current semester and experience with simulations. Questions three to eight consisted of quantitative data along with open-ended comments (Table 1).

**Table 1.** Questions Three to Eight.

Q3: Did you get sufficient information about the manikin, the room, and the equipment before the simulation?
Q4 Did you get sufficient information about the patient cases before the simulation?
Q5: Did you find that this simulation day provided you with something you can use as a future nurse?
Q6: To what degree did you feel that the simulation contributed to increasing your competence in the acute care of injured/ill patients?
Q7: To what degree do you believe the simulation has enhanced your ability to work in a team?
Q8: To what degree did you consider the debriefing session valuable for you?

Finally, questions nine and 10 were open-ended, asking about their overall impression of the day, what went well, and what could be improved.

### Data Analysis

Qualitative data from the open-ended comments were thematically analyzed in six phases (Braun & Clarke, 2021; Herzog et al., 2019). From the beginning, all qualitative data from open-ended questions were analyzed each semester separately to find out if there were differences in the experience of medical simulation between the two semesters. First, the authors read and reread the data to become familiar with it and made brief notes about the analytic insights and ideas to capture important points related to the research question. In phase two, the data were systematically coded to capture relevant single meanings or concepts. During this phase, the two data sets, that is, each semester, were organized into meaningful groups, which means that all data extracts with the same meaning were grouped together. The coding was done manually, and in semester three, 15 codes emerged, while in semester five, 12 codes emerged.

In phase three, initial themes were generated from shared pattern meanings in the dataset. It appeared that the codes from the two semesters were similar, and the decision was made to analyze the data further in the same themes (Table 2).

**Table 2.** Example From the Analysis, Where the Codes From Both Semesters Were Included in the Same Theme.

	Codes	Theme
Semester three	Increased knowledge for acute situations	Enhanced knowledge
	A thorough enhances knowledge	
Semester five	Development of new knowledge	
	Increased knowledge and understanding	
	A possibility for progress	

Phase four involved developing and reviewing themes by going back to the full data set. In cases where the data from the two semesters differed within the themes, it was noted and explained under the theme. In phase five, the themes were defined, refined, and named by revisiting the data to ensure that each theme was built on a strong core concept or essence. Finally, in phase six, the results from phase three onwards were integrated and written down. Here, one overall theme emerged, and therefore, the themes from phase five were renamed as subthemes.

Quantitative data were analyzed as descriptive statistics using SPSS with answers from the close-ended questions and were presented separately for semesters three and five. Crosstabs were used to generate a table presenting the percentages of values in the two variables (Pallant, 2020).

Finally, a mixed-method synthesis was made using an inductive-sequential design with qualitative core components and quantitative supplemental components (Schoonenboom & Burke Johnson, 2017). The themes from the thematic analysis were used as the structure for the mixed analysis, where the results from the quantitative data were used to strengthen the results from the thematic analysis.

### Rigor

To ensure the rigor of the thematic analysis, all steps of the method, as outlined by Braun and Clarke (2006), were followed strictly. The first author is an experienced nurse with extensive expertise in teaching medical simulation and also served as a teacher for certain groups during both semesters included in the study. The second and third authors are teachers at the university but are not involved in teaching medical simulation. They are experienced healthcare researchers, contributing to the trustworthiness of the study. All qualitative data were read and analyzed by the first author, while the other authors reviewed and analyzed a portion of the data. Coding was initially carried out by the first author. Credibility and dependability of the codes and themes were then established through discussions among the authors until a consensus was reached.

The quantitative data were first analyzed separately for each semester. The mixed-method synthesis was then conducted to strengthen the results from the qualitative analysis. In the thematic analysis, the results were initially analyzed separately for each semester but were later combined, as it appeared that the codes from the two semesters were similar. This result was further supported by the quantitative data, which showed no significant differences between the semesters.

It is difficult to assess confirmability and transferability to other contexts, as this study was conducted in relation to medical simulation at a specific university. However, the performance of the simulations is described in detail, and the results are illustrated with quotations to enable the reader to evaluate the credibility of the interpretation and assess the potential transferability to different contexts.

### Ethics

Every student answered the questionnaire anonymously, and the answers could not be traced to any individual. The answers did not contain any personal information. The questions were an evaluation of a significant lesson. The students answered voluntarily, and no one was compelled to answer. This study does not fall under the Swedish Act Concerning Ethical Review of Research Involving Humans (SFS, 2003, p. 460), as there were no risks of hurting or harming any student. Therefore, no ethical permission was needed.

## Results

**Figure 1.**
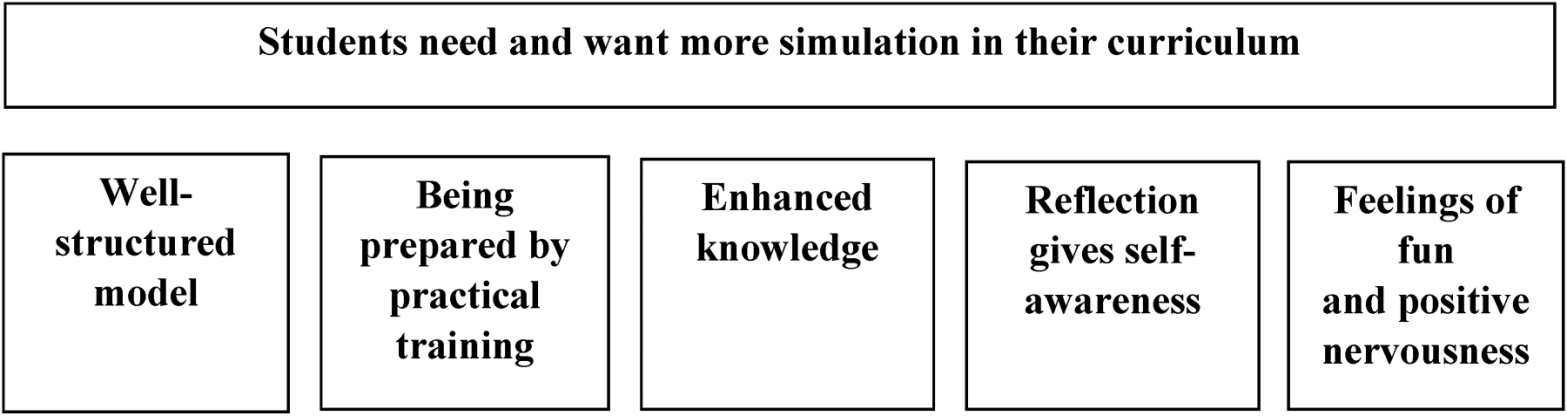
Overall theme and subthemes.

An overall theme in the results emerged from the qualitative data and was divided into five subthemes that described the nursing students’ experiences with medical simulation using both qualitative and quantitative data (Figure 1).

## Students Need and Want More Simulation Training in Their Curricula

Overall, the result revealed that the students generally viewed the simulation training positively. Groups from both semesters noted that it was a useful didactic model. They wanted more simulation training during the whole program, with at least one training session per semester.

We want to have simulation each semester and the whole day instead of a half. (Semester five, student no. 100)

They also wanted more scenarios so that they could be better prepared. Many students responded this way to several of the open-ended questions.

Very valuable to try different roles, work in action, and be an observer. (Semester three student no. 21)

We want more time with simulation. (semester three, student no. 15)

We should have had this more frequently because it is so good! (Semester five, student no.30)

## Well-Structured Model

The students described the medical simulation as a calm and methodical class with a clear structure. They generally had positive opinions about the teachers’ efforts and believed that the simulation instructions were clear and sufficient, with good assistance for reflection afterwards. However, the students felt that the time spent on simulations was too short and sought longer simulation days.

Good overview, pedagogical and well-structured. (Semester three, student no. 55)

Good review, good information, and a well-structured simulation day. (Semester three, student no. 53)

Nice and a welcoming atmosphere. (Semester five, student no. 99)

Overall good, the only negative aspect was the lack of time! (Semester five, student no. 67)

All these quotes are responses to the last two open-ended questions, nine and 10 from both semesters three and five.

The quantitative data also showed that the students in both semesters had received enough information before the simulation training. For question three, “*Did you get enough information about the room and the manikin before the simulation*”*?* 69% of students in semester three and 74% in semester five answered: “*Yes, absolutely.*” Additionally, 31% in semester three and 26% in semester five answered “*Yes, I got enough.*”

For question four, “*Did you get enough information about the patient cases before the simulation*”? 45% of students in semester three and 58% in semester five answered “*Yes, absolutely.*” Furthermore, 51% in semester three and 40% in semester five answered “*Yes, I got enough*,” while 4% in semester three and 2% in semester five answered “*A little, but it was enough.*”

## Being Prepared Through Practical Training

The practical training in the simulation helped the students feel prepared for clinical placement and more prepared to become professional nurses. They learned to integrate multiple aspects of what they have learned as a whole.

I feel more prepared for my upcoming professional life. (Semester five, student no. 62)

Valuable preparations for clinical placement. (Semester three, student no. 1)

Very valuable, perhaps the most important part of the entire education! (Semester five, student no. 3).

Much needed, as it can be difficult to put theory into practice. (Semester three, student no. 52)

**Figure 2.**
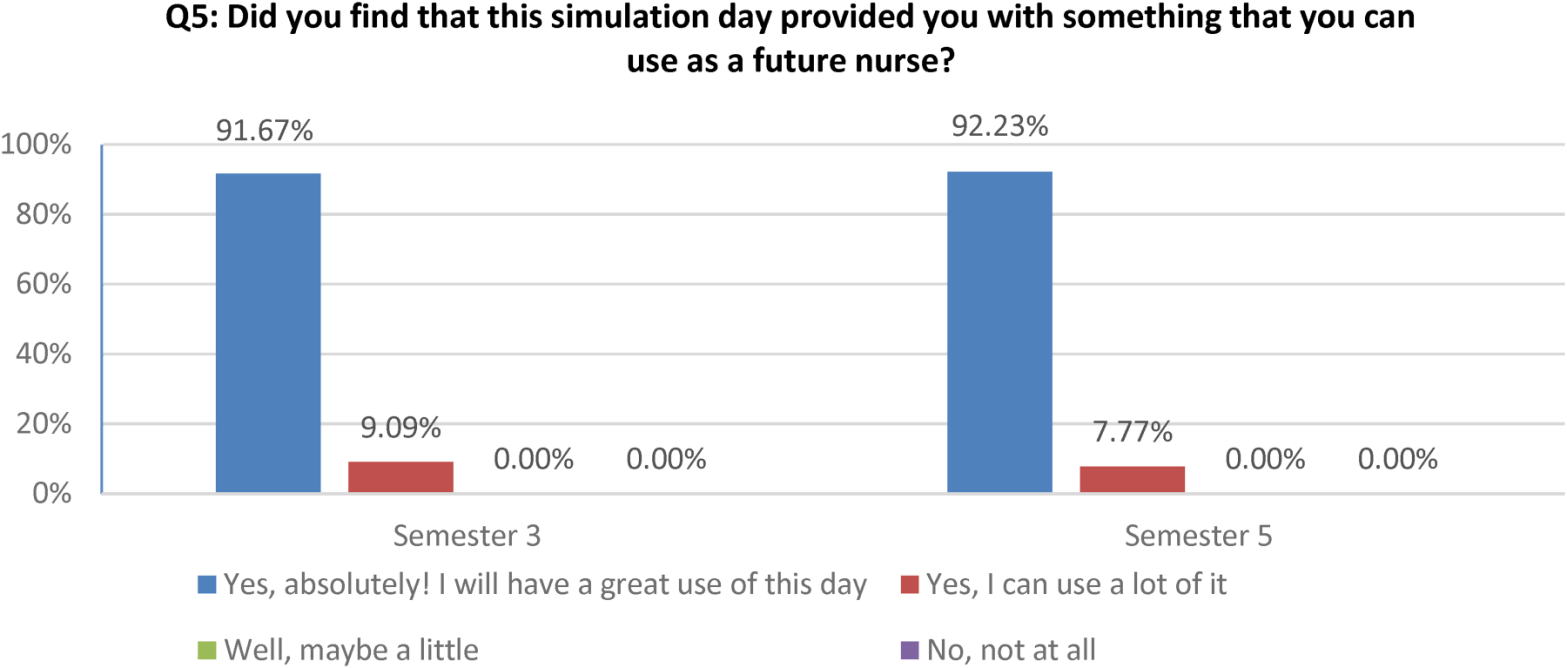
The use of simulation as a future nurse.

Also, the quantitative data shows that over 90% of students in both semesters found the simulation to be greatly useful for the future (Figure 2).

The students in both semesters also expressed that it was valuable to practice teamwork. Many of them believed that good teamwork and collaboration were necessary and found those virtues beneficial to their upcoming profession.

Very valuable to train in a team and practice acute situations that need training. (Semester five, student no. 28)

I've learned a lot about teamwork and communication. (Semester three, student no. 43)

**Figure 3.**
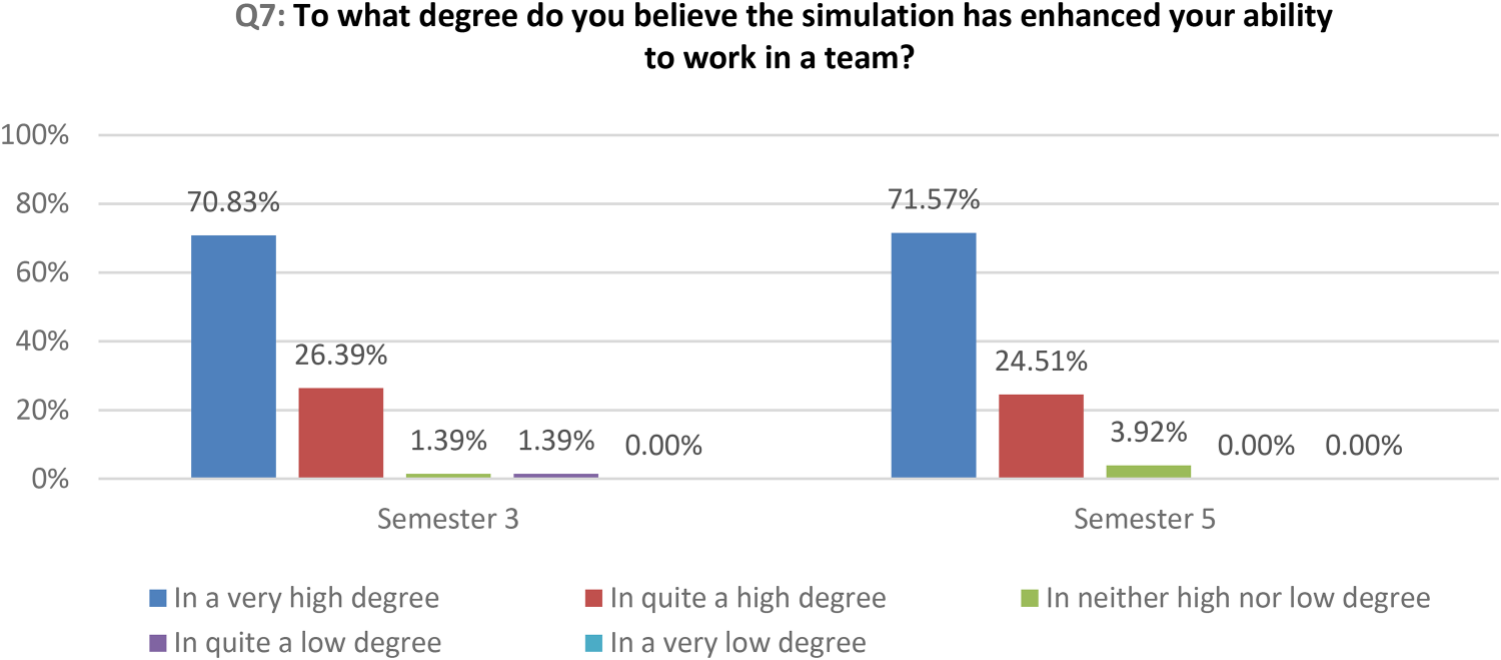
How the simulation has enhanced students’ teamwork skills.

Also, the results of the quantitative data analysis show that students enhanced their ability to work in a team to a very high or in quite a high degree in both semesters (Figure 3).

## Enhanced Knowledge

Students in both semesters noted that the simulation enhanced their knowledge of acute situations. It was important for them to practice in realistic settings, with acute care, using the ABCDE concept and learning that the concept was useable. They also wanted to learn more about acute situations.

I feel more secure to handle in acute crisis and how to cope with my own stress in these situations. (Semester five, student no. 6)

Provided many new insights for acute situations. (Semester five, student no. 13)

I'll take with me how important it is to follow ABCDE. (Semester three, student no. 64)

Very educational day, really important! (Semester three, student no. 61)

Students in both semesters described increased knowledge, understanding, and a possibility for progress, but also understood that they need more training for the future.

I want to learn more in order to have enough knowledge to handle these situations. (Semester five, student no. 53)

Provides me with enhanced knowledge and understanding. (Semester five, student no. 37)

Very educational to practice these skills. (Semester three, student no. 17)

**Figure 4.**
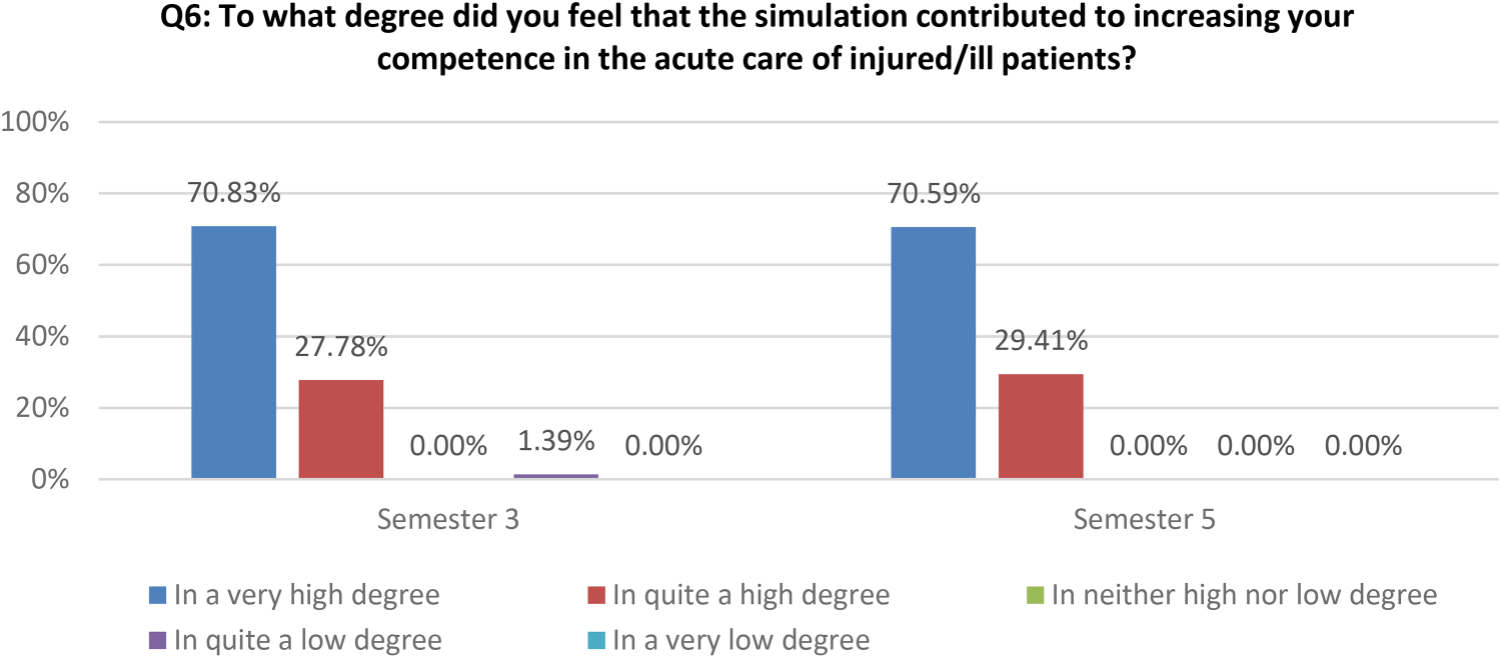
How the simulation increased students’ competence in acute care.

Additionally, the quantitative data show that the simulation contributed to the students’ increased competence in acute care to a very high or quite high degree for both semesters (Figure 4).

### Reflection Gave Self-Awareness

The reflections during the simulation debriefings increased the students’ self-awareness. The students found that the debriefing reflections deeply built their awareness and taught them to reflect on their actions, values, and motivations.

Each situation is unique—as a nurse, one must think so differently, avoid fixation, remain calm, not lose focus and be able to prioritise. (Semester five, student no. 90)

Knowledge of one's own strengths and weaknesses. (Semester three, student no. 63)

**Figure 5.**
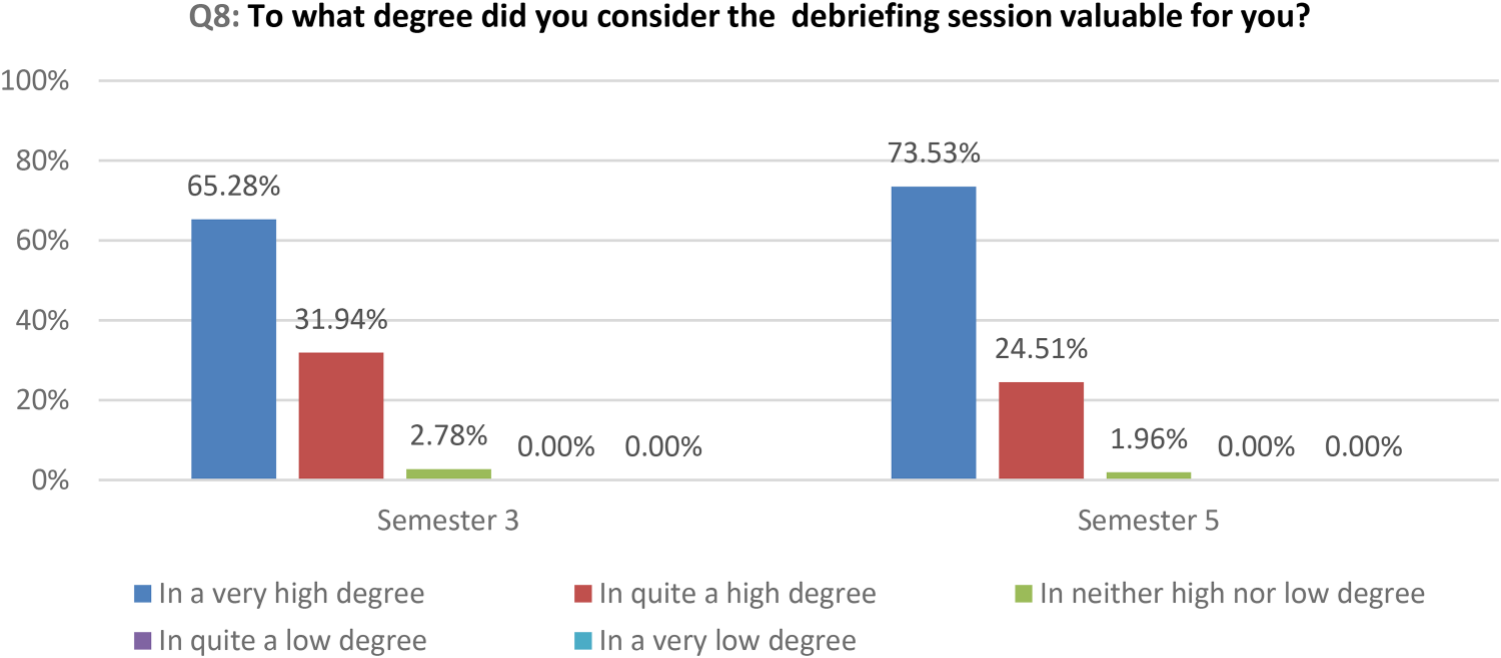
How valuable the debriefing session was to the students.

The quantitative data show that debriefing sessions are valuable for most of the students in a very high or in quite a high degree (Figure 5).

### Feelings of Fun and Nervousness

The simulation was considered fun and amusing, with a positive nervousness. Students often mentioned that finding their own solutions to patients’ problems put them in a good mood. They felt satisfied with their performance with the patients.

From feeling tense and nervous about the moment, it turned out well! (Semester three, student no. 5)

Nervous and tough, but after the simulation, it felt better, both physically and mentally! (Semester five, student no. 20)

Fun, but challenging!; Fun and educational day. (Semester three, student no. 55 and no. 68)

Fun to do, and not just listen, in a situation where it's okay to make mistakes! (Semester five, student no. 39)

## Discussion

The overall theme, “students need and want more simulation in their curriculum,” was developed as the students clearly declared that they need more simulation training. According to Chiniara et al. (2012), the simulation training includes both technical- and nontechnical skills. Even if the result of this study showed that the students experienced that they increased their skills in acute care after the simulation training the results indicated that they needed more training and education to learn, for example, recognition of and situational awareness of sudden patient deterioration. This is in line with the result of Bogossian et al. (2016) who described that undergraduate nursing students may have difficulty recognizing patient deterioration.

The students also indicated that they wanted longer days to have the opportunity to be involved in more scenarios. Sometimes, there is only enough time for a student to participate in one scenario, and they do not get the chance to repeat the exercise and improve. In semester six, the students participate in a full day of medical simulation training with medical students, where they engage in at least three scenarios. They were more satisfied with this length.

Furthermore, the results show that most of the students believe that this day of simulation provides them with valuable insights that can be applied in their future nursing practice. The students mostly noted that the simulation class was valuable for their prospective nursing profession. They also noted that it increased their competence in acute care. This result is in line with Borg Sapiano et al. (2018), who described the effectiveness of medical simulation training in improving nursing students’ competence during patient deterioration. Orique and Phillips (2018) also showed that simulation training helps students recognize clinical deterioration. In a systematic review by Tonapa et al. (2023), the authors recommend incorporating high-fidelity simulation into nursing curricula, as it enables students to function more effectively and efficiently in clinical settings. The students’ knowledge increased, and they gained greater self-confidence.

When working as a professional nurse, various situations arise where quick action is necessary due to deteriorating patients’ conditions. Therefore, medical simulation training is necessary and needs to be used more in nursing education.

The students experienced enhanced competence after medical simulation but wanted more. This is important for the teachers when creating the simulation, as the goal is to develop competence in the form of cognitive skills. These skills can be built upon the student's body of knowledge, which can be improved by setting clear goals for each medical simulation session within the curriculum. Bloom's Taxonomy (Adams, 2015), which distinguishes between six levels of cognitive skills, can be applied here, as other parts of the curriculum, beyond just the simulation, must also be considered. The students in this study had reached the first level, *knowledge*, through lectures on acute care ABCDE and CRM principles prior to the simulation sessions in both semesters three and five. The next level, *comprehension*, is achieved when students can discuss the knowledge in their own words and explain it to others.

The third level, *application*, is represented by the medical simulation part, where students use their knowledge, skills, or techniques in new situations. The fourth level, *analysis*, is also addressed in the simulation, where critical thinking comes into play. In this stage, students must process information and determine how they should act to provide the best care. The fifth level, *synthesis*, is the result of the analysis, where the team creates a care plan. Finally, after the simulation session, the debriefing takes place, which corresponds to the final step of Bloom's Taxonomy, *evaluation*. This step is crucial for the students’ critical thinking development.

The results of this study show that the students felt the need for more medical simulation, as they were not satisfied with their performance in all steps of the simulation, even though they reported increased competence. Here, teachers, when developing the curriculum, must also consider the first two steps of Bloom's Taxonomy, *knowledge* and *comprehension*, to determine whether the preparation before the simulation can be enhanced. The fact that all knowledge dimensions are used in the pedagogical model can be seen as a success factor that influences students’ sense of increased competence, while also helping to further develop the curriculum.

Another interesting result was the positive feelings experienced during the debriefing, which made the students feel more relaxed and gave them opportunities to reflect more easily. Welman and Spies (2016) found that students often felt insecure or anxious before the simulation class. In this study, the students were prepared with a lesson in ABCDE, but they still felt nervous about the manikin's functionality or being watched by other students. Therefore, positive feelings contributed to the positive outcome of the lesson. Positive emotions are also important for learning skills (Dieckmann et al., 2017).

## Strengths and Limitations

This study was conducted at a single university, so the results may not be transferable to all contexts. However, as the performance of the medical simulation at this university is described, the reader can assess the transferability to their own context. The initial purpose of the questionnaire was for teachers to evaluate the implementation of the pedagogical model for further development of the pedagogy and was not validated. However, the questionnaire's questions match questions raised in other literature on the subject, which means that they probably capture the central point of the pedagogic model. As this study began with the analysis of qualitative data, including open-ended questions about the students’ overall impression of the day, the results reflected the entire experience, not just the fixed questions. The results thus captured more, as evidenced by the emergence of a subtheme from the thematic analysis, “*Feelings of fun and nervousness*,” which was not addressed in the questions. Therefore, this study can contribute to further validation of the questionnaire.

The qualitative data consisted of short written responses, whereas interviews could have provided a broader understanding of the students’ experiences. Although, as the students have written a lot of short answers, this part has been seen as important to get a better understanding of their experiences of medical simulation.

## Conclusions

The students experienced increased competence in both technical and nontechnical skills but also recognized the need for additional medical simulation training and expressed a desire for more. They stated that simulation training is beneficial for their future roles as nurses, as it enhances their competence in acute care and improves their teamwork skills. They feel better prepared to handle acute situations through this medical simulation training. Bloom's Taxonomy provides a useful framework for designing and developing the curriculum, as medical simulation plays a crucial role in advancing all levels of cognitive skills and increasing students’ sense of competence.
